# Case Report: Bilateral Epiphysiodesis Due to Extreme Tall Stature in a Girl With a *De Novo DNMT3A* Variant Associated With Tatton-Brown-Rahman Syndrome

**DOI:** 10.3389/fendo.2021.752756

**Published:** 2021-10-13

**Authors:** Otto Lennartsson, Maria Lodefalk, Henrik Wehtje, Eva-Lena Stattin, Lars Sävendahl, Ola Nilsson

**Affiliations:** ^1^ Department of Pediatrics, Örebro University Hospital, Örebro, Sweden; ^2^ Department of Medical Sciences, Örebro University, Örebro, Sweden; ^3^ Department of Orthopedic Surgery, Astrid Lindgrens Children’s Hospital, Karolinska University Hospital, Karolinska Institutet and University Hospital, Stockholm, Sweden; ^4^ Department of Immunology, Genetics and Pathology, Uppsala University, Uppsala, Sweden; ^5^ Division of Pediatric Endocrinology, Department of Women’s and Children’s Health, Karolinska Institutet and University Hospital, Stockholm, Sweden

**Keywords:** DNMT3A, Tatton-Brown-Rahman Syndrome, tall stature, overgrowth, epiphysiodesis surgery

## Abstract

**Objective:**

To present a rare clinical case of a patient with Tatton-Brown-Rahman syndrome and the outcome of tall stature management with bilateral epiphysiodesis surgery at the distal femur and proximal ends of tibia and fibula.

**Study Design:**

Clinical case report.

**Results:**

This is a 20-year-old female with a history of proportional tall stature, developmental psychomotor and language delay with autism spectrum behavior and distinctive facial features. At 12 years and 2 months of age she was in early puberty and 172.5 cm tall (+ 2.8 SDS) and growing approximately 2 SDS above midparental target height of 173 cm (+ 0.9 SDS). A bone age assessment predicted an adult height of 187.1 cm (+3.4 SDS). To prevent extreme tall stature, bilateral epiphysiodesis surgery was performed at the distal femur and proximal ends of tibia and fibula at the age of 12 years and 9 months. After the surgery her height increased by 12.6 cm to 187.4 cm of which approximately 10.9 cm occurred in the spine whereas leg length increased by only 1.7 cm resulting in a modest increase of sitting height index from 50% (-1 SDS) to 53% (+ 0.5 SDS). Genetic evaluation for tall stature and intellectual disability identified a *de novo* nonsense variant in the *DNMT3A* gene previously associated with Tatton-Brown-Rahman syndrome.

**Conclusion:**

Tatton-Brown-Rahman syndrome should be considered in children with extreme tall stature and intellectual disability. Percutaneous epiphysiodesis surgery to mitigate extreme tall stature may be considered.

## Introduction

The genetic cause of Tatton-Brown-Rahman syndrome (TBRS) was first reported in 2014 and is characterized by intellectual disability and tall stature. Other common phenotypes include macrocephaly, autistic spectrum disorder, and distinct facial features (including low-set, heavy, horizontal eyebrows) (1–3). It is caused by pathogenic variants in the *DNMT3A* (DNA methyltransferase 3 alpha) gene, which is a DNA methyltransferase that adds *de novo* methyl groups to cytosine moieties and thus involved in epigenetic regulation of gene expression during development (1). Somatic mutations in *DNMT3A* are also commonly identified in acute myeloid leukemia (4–6). Most of the genetic variants causing TBRS are *de novo*, heterozygous *DNMT3A* missense variants, but microdeletions at 2p23.3 including *DNMT3A* have also been reported (1, 2).

The options available to prevent extreme tall stature in children are limited and includes estrogen (girls) or testosterone (boys) treatment, to advance skeletal maturation and therefore growth cessation, or surgical treatment to physically destroy selected growth plates and thereby limit growth. During percutaneous epiphysiodesis surgery the most accessible growth plates of the legs, ie. growth plates of distal femur, proximal tibia, and proximal fibula, are drilled and curetted to induce bone bridge formation in all quadrants of the growth plates (7) and thereby prevent most of the remaining leg growth. Previous studies have shown that epiphysiodesis surgery performed at the right time have the potential to significantly decrease the remaining growth of the legs and thereby reduce adult height in both males and females (7–9).

We report on the clinical outcome and genetic background of tall stature management with bilateral epiphysiodesis surgery in a girl with TBRS.

## Subject and Methods

The subject of this case report participated in a study cohort for the evaluation of efficacy and safety of percutaneous epiphysiodesis surgery to prevent extreme tall stature (7), and a study of genetic causes for short and tall stature that was approved by the Swedish Ethical Review Authority, Sweden, (reference no. 2015/1787-31; 2018/581-32; 2021-01694) and written informed consent was obtained from the subject and her parents. Height, weight, head circumference were plotted on the Swedish growth charts, and z-scores were calculated using the Swedish growth reference data (10). For sitting height and sitting height index, the reference of Fredriks AM et al., was used (11). The evaluation of puberty was assessed according to the methods described by Tanner (12), while bone age was assessed by two independent readers according to the Greulich and Pyle method (13).

### Case Report

This is a 20-year-old woman with overgrowth, intellectual disability, macrocephaly and marked, horizontal eyebrows born to unrelated, healthy parents of normal stature; mother’s height is 176 cm (+1.4 SDS) and father’s height is 183 cm (+0.4 SDS). She was born at 40 weeks of gestation after an uncomplicated pregnancy with a birth weight of 4.4 kg (+ 0.9 SDS) and birth length of 52 cm (+ 1.0 SDS). During her first years of life, she was growing slightly faster than normal, continuously gaining height percentiles. At 3 years and 3 months of age her height was 113.5 cm (+2.9 SDS). At 7 years of age she underwent extensive neuropsychiatric evaluation that resulted in a diagnosis of autism spectrum disorder.

At the age of 12 years and 2 months she was in early puberty (Tanner stage B2), her height was 172.5 cm (+2.8 SDS) and bone age according to Greulich and Pyle was 12 years resulting in a predicted adult height of 187.1 cm (+3.4 SDS) according to the Bayley-Pineau tables (14). At the age of 12 years and 9 months, she underwent bilateral percutaneous epiphysiodesis surgery at the distal femur, proximal tibia and proximal fibula bilaterally due to a concern of extreme tall stature. The surgical method has been described earlier (7).

### Sequencing

Genomic DNA was obtained from a blood sample of the subject. Given the specific phenotype, the sample was sent to Invitae corporation (San Francisco, CA, USA) for analysis of 21 genes associated with overgrowth and intellectual disability (**Table 1**). Briefly, DNA was enriched for target regions using hybridization-based protocol and thereafter sequenced using the Illumina technology platform. All target regions were sequenced with > 50x depth. Reads were aligned to a reference sequence (GRCh37) and sequence changes were identified and interpreted in the context of a single clinically relevant transcript. Enrichment and analysis focus on the coding sequence of the individual transcripts, 10bp of flanking intronic sequence and other specific genomic regions demonstrated to be causative of disease at the time of assay design. Exonic deletions and duplicates are called using an in-house algorithm that determines copy number at each target comparing the read depth for each target in the proband sequence with both mean read depth and read depth distribution obtained from a set of clinical samples.

**Table 1:** The complete list of genes in the gene panel for tall stature analyzed during sequencing provided by Inviate Corporation^©^.

Gene	Transcript
AKT2	NM_001626.5
AKT3	NM_005465.4
CDKN1C	NM_000076.2
CUL4B	NM_003588.3
DIS3L2	NM_152383.4
DNMT3A	NM_175629.2
EZH2	NM_004456.4
GLI3	NM_000168.5
GPC3	NM_004484.3
KPTN	NM_007059.3
MED12	NM_005120.2
MTOR	NM_004958.3
NF1	NM_000267.3
NFIX	NM_001271043.2
NPR2	NM_003995.3
N5D1	NM_022455.4
PHF6	NM_032458.2
PIK3R2	NM_005027.3
PTEN	NM_000314.4
SETD2	NM_014159.6
SPRED1	NM_152594.2

## Results

### Percutaneous Epiphysiodesis Surgery

**Figure 1:**
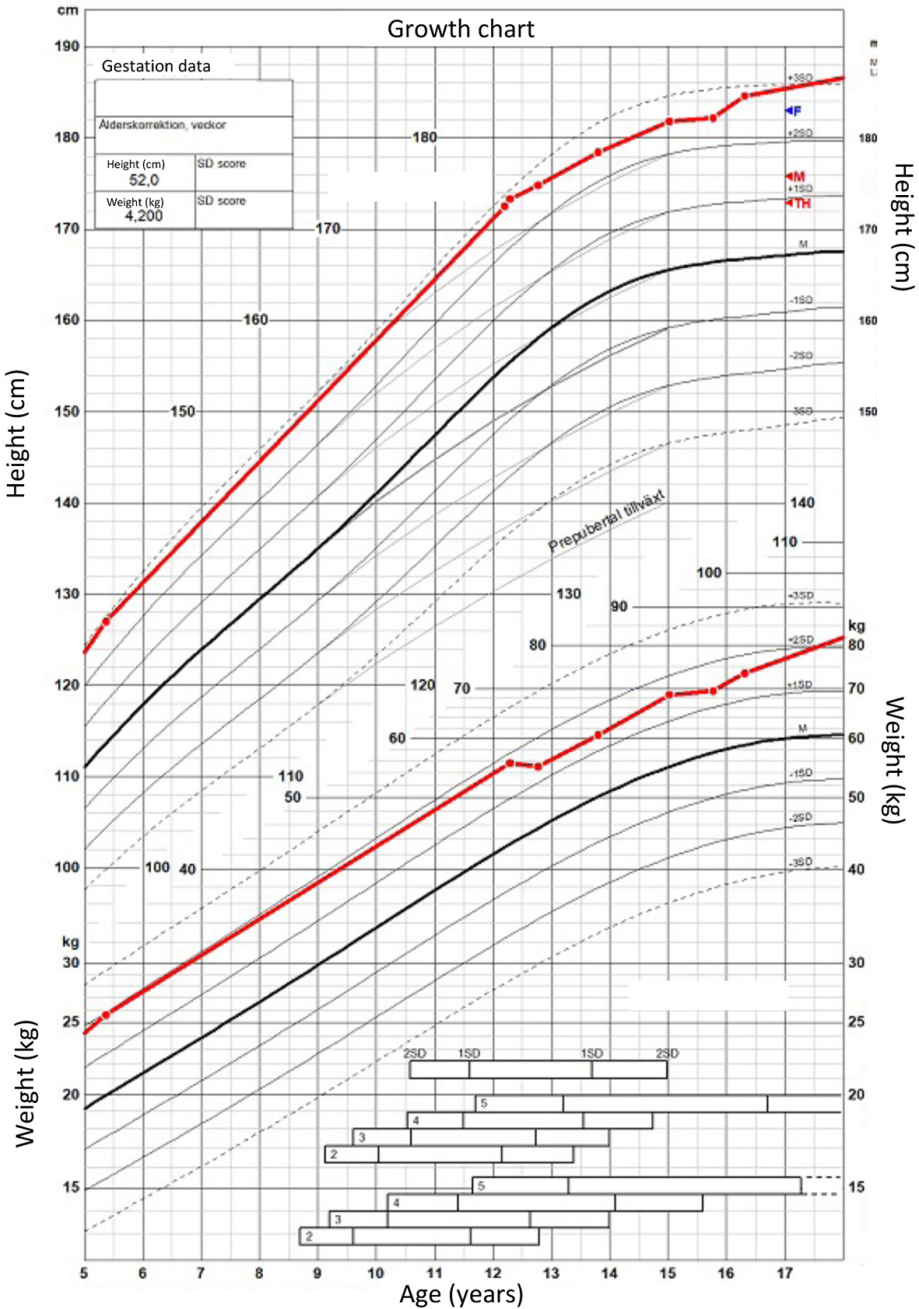
Height and weight growth chart from 5 to 18 years of age for the proband.

**Figure 2:**
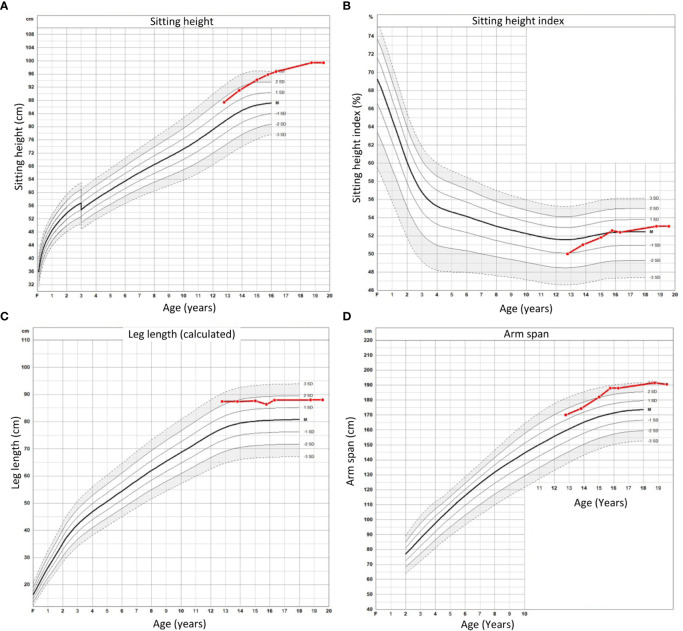
Sitting height **(A)**, sitting height index **(B)**, calculated leg length **(C)**, and arm span **(D)** for proband from time of percutaneous epiphysiodesis surgery to final height.

Percutaneous epiphysiodesis surgery was performed at the age of 12 years and 9 months in order to stop growth at the growth plates of distal femur, proximal tibia and proximal fibula bilaterally. The surgery was uncomplicated and she was discharged from the hospital the day after the surgery with full weight bearing on crutches for 2 weeks. At the point of percutaneous epiphysiodesis surgery her height was 174.8 cm (+ 2.7 SDS: **Figure 1**), sitting height 87.4 cm (+ 1.7 SDS; **Figure 2A**), sitting height index 50% (- 1.0 SDS; **Figure 2B**), leg length by x-ray was measured at 89.4 cm (right) and 88.1 cm (left), calculated leg length (height - sitting height) was 87.4 cm (+ 2.3 SDS; **Figure 2C**), and arm span 170 cm (+ 1.4 SDS; **Figure 2D**). Final adult height was assessed at the age of 19 years and 6 months at which point her height had remained the same for the last 10 months and only increased 2.8 cm in the last 3 years (since the age of 16 years and 3 months) (**Figure 1**). From the epiphysiodesis surgery her height had increased with 12.6 cm resulting in an adult height of 187.4 cm (+ 3.2 SDS). Most of this growth had occurred in the spine as sitting height had increased 10.9 cm to 99.4 cm (+ 3.9 SDS; **Figure 2A**) during the same time period, resulting in a modest increase of her sitting height index from 51% (-1.0 SDS) to 53% (+ 0.5 SDS; **Figure 2B**). Leg length by x-ray had increased 1.7 cm to 91.1 cm and 3.3 cm to 91.4 cm for right and left leg, respectively, whereas calculated leg length had increased 1.7 cm, from 87.4 cm (+ 1.6 SDS) to 89.1 cm (+ 1.4 SDS). In contrast, arm span had increased 20.5 cm to 190.5 cm (+ 3.0 SDS) at adult height (age 19 years and 6 months). Legs remained straight with no clinically significant or radiological genu varum or valgum. Standing long leg X-ray angles had decreased from approximately 3.0 degrees bilaterally before surgery to 0.9 and 1.7 degrees at the left and right knee, respectively, at final height.

### Sequencing

Targeted sequencing identified a rare heterozygous nonsense variant in *DNMT3A* (NM_175629.7): c.958C>T (p.Arg320*) in the proband, but not in either of her parents. The sequence change creates a premature stop codon in the *DNMT3A* gene predicted to result in an absent or disrupted protein product. This variant has previously been associated with TBRS in one patient (3) and is described as pathogenic regarding TBRS in ClinVar (15). According to GnomAD, it exhibits an allele frequency of 0.00001769 (16).

## Discussion

The patient underwent percutaneous epiphysiodesis due to a concern of extreme tall stature. Predicted final height based on bone age exam resulted in a final height of 187.1 cm (+ 3.4 SDS) whilst her actual adult height was similar at 187.4 cm. Similarities between her predicted final height and her adult height despite epiphysiodesis surgery could potentially be due to the lack of effect of the surgery. However, this is unlikely as the growth plates are identified and the position of the instruments are continuously monitored using an X-ray image intensifier (C-arm) and the surgery was performed by an experienced surgeon. A more likely explanation is that the height prediction underestimated the amount of remaining growth. Height predictions are based on normal populations with very few numbers and thus weak statistical power at the extremes and are not validated for individuals with specific growth syndromes. This interpretation is supported by the finding that the legs only grew less than 2 cm after the surgery whereas sitting height increased by more than 10 cm and arm span increased by more than 20 cm. In other words, if her legs would have grown as much as her arms, ie. 10 cm each, after the surgery, she would have reached an adult height of 195 cm or more.

The c.958 C > T nonsense variant in *DNMT3A* found in the proband has previously been reported in association with TBRS in the ClinVar database and also been reported in the Catalogue of somatic Mutations in Cancer (COSMIC) database (3). It introduces a premature stop codon, i.e., a nonsense variant, and is predicted to result in a truncated protein and is pathogenic according to FATHMM prediction. This variant likely results in a non-functional protein product, likely rendering the patient functionally haploinsufficient for *DNMT3A*. DNMT3A is a methyltransferase responsible for *de novo* methylation of cytosines in CpG dinucleotides, which is an epigenetic mark associated with repression of gene expression (17). Therefore, it may be speculated that the mechanism of overgrowth in TBRS involves derepression and thus increased expression of growth-promoting genes in growth plate chondrocytes. This would be a mechanism similar to the one we previously proposed for overgrowth in Weaver syndrome (18).

In summary, we report a young female with a rare, *de novo* variant in *DNMT3A* and TBRS who underwent percutaneous epiphysiodesis surgery that successfully limited the remaining growth of her legs. These findings support the pathogenicity of the c.958C>T (p.Arg320*) variant in TBRS, suggest that adult height predictions may underestimate the remaining growth in individuals with TBRS, and that percutaneous epiphysiodesis surgery may be considered in individuals with TBRS and other overgrowth syndromes in order to mitigate extreme tall stature.

## Data Availability Statement

All data included in this study are available from the corresponding author upon request.

## Ethics Statement 

The studies involving human participants were reviewed and approved by Etikprövningsmyndigheten, Uppsala, Sweden. The patients/participants provided their written informed consent to participate in this study.

## Author Contributions

OL retrieved data from the medical records, generated the table, and wrote the first draft of the manuscript. ON was responsible for the study design and the interpretation of the findings, generated the figures, retrieved data from the medical records and edited all versions of the manuscript. ML, E-LS, HW, and LS contributed expertise in diagnosis and management of tall stature syndromes and read and edited the manuscript. All authors contributed to the article and approved the submitted version.

## Funding

This work was supported by grants from the Swedish Research Council (project K2015–54X-22 736–01–4 & 2015-02227), the Stockholm County Council, Novo Nordisk Foundation, Erik och Edith Fernström Foundation for Medical Research, HKH Kronprinsessan Lovisas förening för barnasjukvård, Sällskapet Barnavård, Stiftelsen Frimurare Barnhuset i Stockholm, Promobilia, Sällsynta Fonden, Nyckelfonden, and Karolinska Institutet, Stockholm, Sweden, and Örebro University, Örebro, Sweden.

## Conflict of Interest

ON has received consulting fees from Kyowa Kirin and Biomarin and speakers’ honoraria from Merck, Pfizer, Kyowa Kirin, Biomarin and research funds to the department of ON from Kyowa Kirin and Novo Nordisk Foundation.

The remaining authors declare that the research was conducted in the absence of any commercial or financial relationships that could be construed as a potential conflict of interest.

## Publisher’s Note

All claims expressed in this article are solely those of the authors and do not necessarily represent those of their affiliated organizations, or those of the publisher, the editors and the reviewers. Any product that may be evaluated in this article, or claim that may be made by its manufacturer, is not guaranteed or endorsed by the publisher.
